# Clinicopathological and prognostic significance of PD-L1 expression in colorectal cancer: a meta-analysis

**DOI:** 10.1007/s00384-020-03734-4

**Published:** 2020-09-10

**Authors:** Shuxia Wang, Bo Yuan, Yun Wang, Mingyang Li, Xibo Liu, Jing Cao, Changtian Li, Jihong Hu

**Affiliations:** 1 Public Health School, Gansu University of Chinese Medicine, Lanzhou, 730000 China; 2 Basic Medical School, Gansu University of Chinese Medicine, Lanzhou, 730000 China; 3 Center of Research and Experiment, Gansu University of Chinese Medicine, Lanzhou, 730000 China

**Keywords:** Programmed death ligand-1 (PD-L1), Colorectal cancer (CRC), Prognosis, Clinicopathological features, Meta-analysis

## Abstract

**Purpose:**

To systematically evaluate the correlation between PD-L1 expression and clinicopathological features and prognosis of colorectal cancer (CRC).

**Methods:**

Seven databases (PubMed, Cochrane Library, EMBASE, Web of Science, CBM, Wanfang, and CNKI) were searched through May 2020. Risk of bias and quality of evidence were assessed by using the Newcastle–Ottawa scale (NOS), and meta-analysis was carried out by using the Review Manager 5.3 software on the studies with the quality evaluation scores ≥ 6. Meta-regression analysis was used to determine the independent role of PD-L1 expression on CRC prognosis after adjusting clinicopathological features and treatment methods.

**Results:**

A total of 8823 CRC patients in 32 eligible studies. PD-L1 expression was correlated with lymphatic metastasis (yes/no; OR = 1.24, 95% CI (1.11, 1.38)), diameter of tumor (≥ 5 cm/< 5 cm; OR = 1.34, 95% CI (1.06, 1.70)), differentiation (high–middle/low; OR = 0.68, 95% CI (0.53, 0.87)), and vascular invasion (yes/no; OR = 0.80, 95% CI (0.69, 0.92)). PD-L1 expression shortened the overall survival (hazard ratio (HR) = 1.93, 95% CI (1.66, 2.25)), disease-free survival (HR = 1.76, 95% CI (1.50, 2.07)), and progression-free survival (HR = 1.93, 95% CI (1.55, 2.41)). Meta-regression showed that PD-L1 expression played a significant role on poor CRC OS (HR = 1.95, 95% CI (1.92, 3.98)) and disease-free survival (HR = 2.14, 95% CI (0.73, 4.52)).

**Conclusion:**

PD-L1 expression independently predicted a poor prognosis of CRC.

**Electronic supplementary material:**

The online version of this article (10.1007/s00384-020-03734-4) contains supplementary material, which is available to authorized users.

## Introduction

Colorectal cancer (CRC) is one of the most common malignant tumors of the digestive system all around the world [1]. Its incidence and mortality rate ranked third and second in the world, respectively [2]. In 2018, both new cases and deaths were close to 30% of the total number of CRC cases in the world [3, 4]. China’s cancer statistics indicated that the incidence and mortality of CRC ranked fifth among all malignant tumors in China, bringing about 380,000 new cases and 190,000 deaths annually [5]. Furthermore, most patients have already been in the severe stage when they were seeking the medical examination [6, 7]. Thus, it has become a major public health problem in many countries [8, 9].

Surgery, chemotherapy, and radiation therapy are the main treatments for cancer; unfortunately, the recurrence rate and metastasis rate (approximately 30% and 10%) in advanced CRC patients still remain high [10, 11]. In addition, some treatments showed only mild effects in reducing tumor load, such as cytokine therapy, toll-like receptors, and autologous cell therapy [12]. In recent years, immune card control point drugs have provided a new therapy for CRC, especially the programmed death 1 (PD-1)/programmed death ligand-1(PD-L1) monoclonal antibody as an immunodetection point inhibitor and an antibody-type tumor immune drug [13, 14]. PD-L1, also known as CD274 or B7-H1, is the ligand PD-1 and a sort of immune checkpoint inhibitors and belongs to the CD28 family and is expressed on the surface of activated T cells to regulate proliferation and activation [15]. The binding of PD-L1 on tumor cells to PD-1 on lymphocytes can lead to immune escape of tumor cells and ultimately promote the generation and development of tumors by inhibiting the release of cytokines, restricting lymphocyte function, and inducing lymphocyte apoptosis [16]. It was reported that PD-L1 correlated with the clinicopathological features and affected the prognosis of cancers (such as breast, gastric, and ovarian cancers) [17–19].

The correlation between PD-L1 expression and clinicopathological features of CRC was inconsistent, and the independent impacts of PD-L1 expression on CRC prognosis were unclear in the previous meta-analyses [20–23]. Additionally, some limitations reduced the reliability because of small sample sizes [21, 23] or the high heterogeneity [21, 23] or incorrect model selection [21, 23]. Thus, we aimed to update a meta-analysis of cohort studies to confirm the correlation between PD-L1 expression and clinicopathological features, and perform a meta-regression analysis to determine the independent role of PD-L1 on CRC prognosis after adjusting confounders.

## Materials and method

### Search strategy

Seven databases (PubMed, Cochrane Library, EMBASE, Web of Science, CBM, Wanfang, and CNKI) were searched through May 2020, and the search strategies were (“PD-L1” OR” B7-H1” OR “Programmed Cell Death Ligand 1” OR “CD274” OR “PD-1” OR “Programmed death 1”) AND (“Colorectal Cancer” OR “Colorectal Neoplasm” OR “Colorectal Tumor” OR “Colorectal Carcinoma” OR “Colorectal Cancer” OR “Rectal Cancer” OR “Colon Cancer” OR “Rectal Neoplasm” OR “Colon Neoplasm”). Furthermore, we reviewed the reference list of original and review articles to search for more studies. Only studies that were published as full articles and in Chinese and English were considered.

### Inclusion and exclusion criteria

Inclusion criteria for study enrollment were (1) cohort studies; (2) patients had confirmed colorectal cancer; (3) PD-L1 expression detected method: immunohistochemistry (IHC); (4) the literature provides the relationship between PD-L1 expression and clinicopathological features, such as sex, age, lymphatic metastasis, differentiation, TNM stage, and tumor location; (5) studies that provided detailed pathological parameters and survival outcomes; and (6) studies that provided hazard ratios and 95% confidence interval (CI) to calculate survival outcomes. The exclusion criteria were (1) studies that were case reports, reviews, or conference papers; (2) republished literature, reviews, and case series; and (3) full text not available.

### Data extraction

Two researchers (Shuxia Wang and Yun Wang) identified and classified the literature that met the inclusion criteria independently and excluded the study that obviously did not meet the inclusion criteria after reading the full text. For studies with insufficient information, we contacted the primary authors to acquire and verify data when possible. In cases of disagreement, the two researchers can make an attempt to reach a consensus. We extracted these objective data which were analyzed for aims of this study : (1) the basic information of the study including first author, year of publication, country, number of subjects, their demographic features, (2) type of study, (3) treatment method, (4) outcomes including the pathological parameters (sex, age, tumor location, TNM stage, lymphatic metastasis, differentiation, infiltration degree, tumor diameter, distant metastasis, and vascular invasion), and (5) prognostic values including overall survival (OS), disease-free survival (DFS) and progression-free survival (PFS).

### Quality assessment

Study quality was assessed by using the Newcastle–Ottawa score [24], which consists of three factors: patients selection, comparability of study groups, and assessment of outcomes. A score of 0 to 9 was assigned to each study, and studies achieving a score of 6 or higher were considered high quality.

### Statistical analysis

If the numbers of included studies were less than 3, the meta-analysis could not be used. All statistical analyses were conducted by using Review Manager 5.3. Odds ratios (OR) and 95% CI were analyzed for the relationship between PD-L1 expression and basic clinicopathological features including sex (male/female), age (≥ 60/< 60 years old), tumor location (right + rectum/left + colon), TNM stage (III–IV/I–II), lymphatic metastasis (yes/no), differentiation (high–middle/low), tumor diameter (≥ 5 cm/< 5 cm), vascular invasion (yes/no), infiltration degree (3–4/1–2), and distant metastasis (yes/no). Hazard ratio (HR) and its 95% CI were presented for PD-L1 on CRC prognosis. Subgroup analysis was used to find the source of heterogeneity according to treatment methods (surgery or surgery combined with chemoradiotherapy (CRT)). Moreover, meta-regression analysis was used to analyze the independent role of PD-L1 on the prognosis of CRC after adjusting for above clinicopathological features and treatment methods. If the numbers of included studies were less than 10, the meta-regression could not be used. Depending on the results from the tests of heterogeneity, a fixed effect model or a random effect model was chosen. The chi-square test and *I*^2^ were used to evaluate the heterogeneity of the included studies. Begg’s test was used to analyze publication bias by using the software Stata, version 15.1.

## Results

### Description of studies and quality assessment

**Fig. 1:**
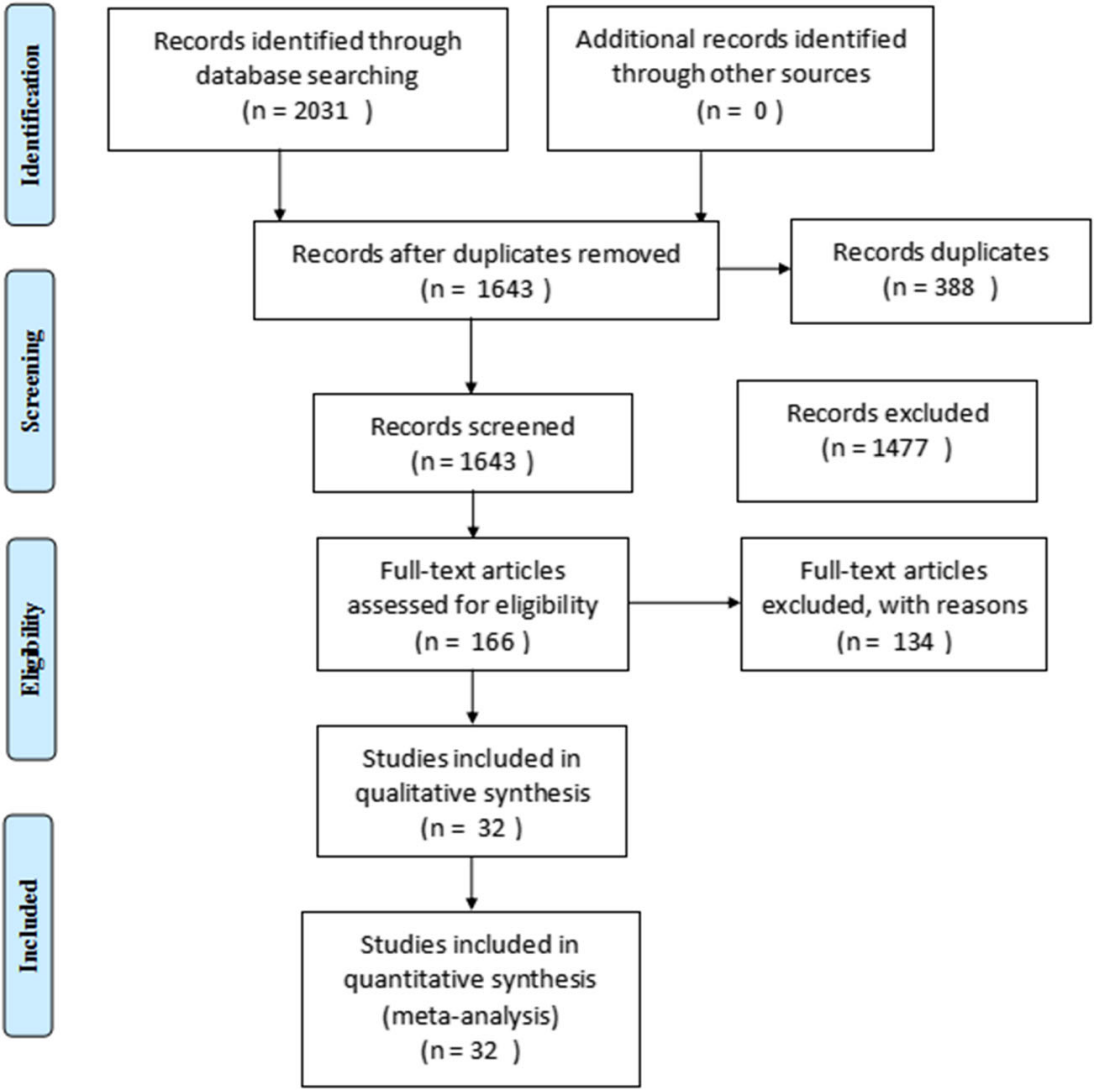
Flowchart of document retrieval

Thirty-two eligible studies [25–56] with Newcastle–Ottawa scale (NOS) score ≥ 6 were included in meta-analysis, including five in Chinese and twenty-seven in English, with a total of 8823 CRC patients. The follow-up duration was from 4 months to 7.3 years, and the sample size was from 65 to 1414. The selection process of literature is detailed in Fig. 1. Basic information and quality evaluation of included studies are presented in Table 1 and Table 2.

**Table 1:** Basic study characteristics

First author	Year	Country	Sample	Age (years)	Sex (male/female)	Type	TNM stage	Treatment	Detection methods for PD-L1 expression	Follow-up duration (months)	Outcome	HR and 95% CI	Cutoff
A Ogura	2018	Japan	287	61 (27–81)	195/92	RC	I–IV	Surgery + CRT	IHC	NA	DFS	1.999 (0.846, 4.725)	Score ≥ 2 (intensity)
Bae SU	2018	Korea	175	68 (35.5–93)	105/70	CRC	I–IV	Surgery + CRT	IHC	88	DFS, OS	1.274 (0.852, 1.904), 1.586 (1.069, 2.353)	> 50% positive cells
Berntsson J	2018	Sweden	526	50–85	247/279	CRC	I–IV	Surgery	IHC	60	OS	0.530 (0.390, 0.720)	≥ 1% TC staining
CY Huang	2018	China	864	NA	463/401	CC	II–III	Surgery	IHC	NA	DFS	1.370 (1.080, 1.740)	≥ 5% TC staining
Droeser RA	2013	Switzerland	1420	69.9 (30–96)	673/741	CRC	I–IV	Surgery	IHC	NA	OS	0.920 (0.880, 0.960)	NA
Enkhbat T	2018	China	116	69.7 (41–93)	77/37	CRC	II–III	Surgery + CRT	IHC	60	DFS, OS	1.913 (0.811, 4.516), 3.873 (1.193, 12.571)	Score > 3 (area and intensity)
HQ Li	2017	China	90	70 (24–90)	47/43	CRC	I–IV	Surgery	IHC	NA	OS	3.180 (1.642, 6.156)	NA
H Zhu	2015	China	120	NA	71/49	SAC	I–IV	Surgery	IHC	NA	OS	0.692 (0.277, 1.729)	Score > 4 (area and intensity)
Hao Jiang	2020	China	65	60 (23–79)	37/28	CRC	I–IV	Surgery	IHC	35	DFS, OS	2.914 (1.307, 4.697), 4.267 (1.144, 15.917)	NA
J Xu	2016	China	97	NA	52/45	RC	I–IV	Surgery	IHC	60	PFS	1.645 (0.809, 3.346)	NA
JY He	2017	China	120	54 (22–87)	81/39	CRC	I–IV	Surgery + CRT	IHC	36	PFS, OS	0.863 (0.333, 2.235), 1.095 (0.382, 3.143)	NA
Koganemaru S	2017	Japan	235	63 (32–84)	140/95	CRC	II–III	Surgery + CRT	IHC	72	DFS	2.450 (1.239, 4.847)	≥ 5% TC staining
L Wang	2019	China	110	55 (26–85)	72/38	CRC	I–IV	Surgery	IHC	24	PFS	NA	NA
Lee KS	2018	Korea	336	63.1 ± 12.5	201/135	CC	I–III	Surgery + CRT	IHC	52	DFS, OS	3.504 (1.461, 8.406), 3.785 (1.447, 9.898)	NA
Lee LH	2016	USA	395	55 ± 15	201/194	CRC	I–IV	Surgery + CRT	IHC	55	DFS	3.600 (1.080, 12.000)	NA
Lee SJ	2018	Korea	89	73 (26–89)	58/31	CC	I–IV	Surgery	IHC	60	DFS	0.325 (0.108, 0.794)	NA
LS Wang	2016	China	262	28–75	166/96	CRC	II–III	Surgery	IHC	60	PFS	1.830 (1.090, 3.050)	NA
M Ahtiainen	2019	Finland	242	NA	62/132	CRC	I–IV	Surgery	IHC	60	DFS, OS	1.200 (0.350, 4.040), 1.180 (0.630, 2.230)	Score ≥ 2 (intensity)
M Song	2013	China	247	NA	160/187	CRC	I–IV	Surgery	IHC	30	OS	2.070 (1.342, 3.193)	≥ 5% TC staining
M Liang	2014	China	185	52 (29–72)	87/98	CRC	I–IV	Surgery	IHC	60	DFS, OS	1.831 (1.214, 2.806), 1.740 (1.195, 2.713)	NA
SJ Shi	2013	China	143	59.8 ± 12.4	61/82	CRC	I–IV	surgery	IHC	60	PFS, OS	2.771 (1.048, 2.994)	NA
S Saigusa	2016	Japan	90	64 (33–80)	64/26	RC	I–IV	Surgery + CRT	IHC	6–12	PFS, OS	3.311 (1.444, 7.591), 2.278 (1.034, 5.016)	NA
ShuFen Chiang	2018	USA	104	59.3 ± 12.5	33/71	RC	I–IV	Surgery + CRT	IHC	4–6	DFS, OS	2.550 (1.050, 6.200), 2.370 (0.760, 7.3800	NA
Takato Yomoda,	2018	Japan	132	NA	67/65	CRC	I–IV	Surgery + CRT	IHC	60	DFS, OS	3.320 (1.170, 11.800), 2.710 (2.720, 2.820)	NA
X Lei	2018	China	80	52.5 (32–77)	40/40	CRC	I–IV	Surgery	IHC	20	PFS	1.587 (1.050, 2.988)	NA
X Gao	2017	China	85	58.6 (23–90)	39/46	CC	I–IV	Surgery	IHC	48	PFS	0.503 (0.254, 0.997)	NA
XL Wei	2018	China	422	56 (24–83)	249/173	CRC	I–IV	Surgery	IHC	72	DFS, OS	0.420 (0.250, 0.720), 0.810 (0.530, 1.230)	≥ 1% IC and/or ≥ 5% TC staining
Yohei Masugi	2016	USA	823	69.1 ± 9.0	365/458	CRC	I–IV	Surgery + CRT	IHC	24	OS	1.020 (0.720, 1.430)	> 50% positive cells
Y Li	2016	China	356	57 (27–85)	199/157	CRC	NA	Surgery	IHC	13	DFS, OS	1.048 (0.639, 1.719), 0.626 (0.332, 1.181)	> 50% positive cells
ZF Xiong	2018	China	250	52 (18–88)	143/107	CRC	I–IV	Surgery	IHC	18	PFS	1.587 (1.050, 2.988)	NA
Z Li	2018	China	153	63 (26–89)	77/76	CRC	I–IV	Surgery	IHC	60	DFS, PFS	3.180 (1.642, 6.156)	≥ 5% TC staining
Zhaoying Wu	2019	China	204	65.5 (25–89)	124/80	CRC	I–IV	Surgery + CRT	IHC	22	OS	1.914 (1.031, 3.553)	NA

*CRC*, colorectal cancer; *SAC*, serrated adenocarcinoma; *CC*, colon cancer; *RC*, rectal cancer; *CRT*, chemoradiotherapy; *IHC*, immunohistochemistry; *NA*, not available; *OS*, overall survival; *DFS*, disease-free survival; *PFS*, progression-free survival; *HR*, hazard ratio; *CI*, confidence interval

**Table 2:** Methodological quality evaluation of included studies by using the NOS

First author	Published year	Sample selection			Comparability?	Outcome			NOS score
		Case definition adequate?	Representativeness of the cases?	Ascertainment of exposure	Comparability?	Assessment of outcome?	Was follow-up long enough for outcomes to occur?	Description of follow-up?	
A Ogura	2018	+	+	+	+	+	+	−	6
Bae SU	2018	+	+	+	+	+	+	+	7
Berntsson J	2018	+	+	+	+	+	+	+	7
CY Huang	2018	+	+	+	+	+	+	+	7
Droeser RA	2013	+	+	+	+	+	+	+	7
Enkhbat T	2018	+	+	+	+	+	+	+	6
HQ Li	2017	+	+	+	+	+	−	−	6
H Zhu	2015	+	+	+	+	+	+	−	6
Hao Jiang	2020	+	+	+	+	+	+	+	7
J Xu	2016	+	+	+	+	+	+	+	7
JY He	2017	+	+	+	+	+	+	+	7
Koganemaru S	2017	+	+	+	+	+	+	+	7
L Wang	2019	+	+	+	+	+	+	+	7
Lee KS	2018	+	+	−	+	+	+	+	6
Lee LH	2016	+	+	+	+	+	+	+	7
Lee SJ	2018	+	+	+	+	+	+	+	7
LS Wang	2016	+	+	+	+	+	+	+	7
M Ahtiainen	2019	+	+	+	+	+	+	+	7
M Song	2013	+	+	+	+	+	+	+	7
M Liang	2014	+	+	+	+	+	+	+	7
SJ Shi	2013	+	+	+	+	+	+	+	7
S Saigusa	2016	+	+	+	+	+	+	+	7
ShuFen Chiang	2018	+	+	+	+	+	+	+	7
Takato Yomoda	2018	+	+	+	+	+	+	+	7
X Lei	2018	+	+	+	+	+	+	+	7
X Gao	2017	+	+	+	+	+	+	+	7
XL Wei	2018	+	+	+	+	+	+	+	7
Yohei Masugi	2016	+	+	+	+	+	+	+	7
Y Li	2016	+	+	+	+	+	+	+	7
ZF Xiong	2018	+	+	+	+	+	+	+	7
Z Li	2018	+	+	+	+	+	+	+	7
Zhaoying Wu	2019	+	+	+	+	+	+	+	7

### Correlation between PD-L1 expression and clinicopathological features

**Fig. 2:**
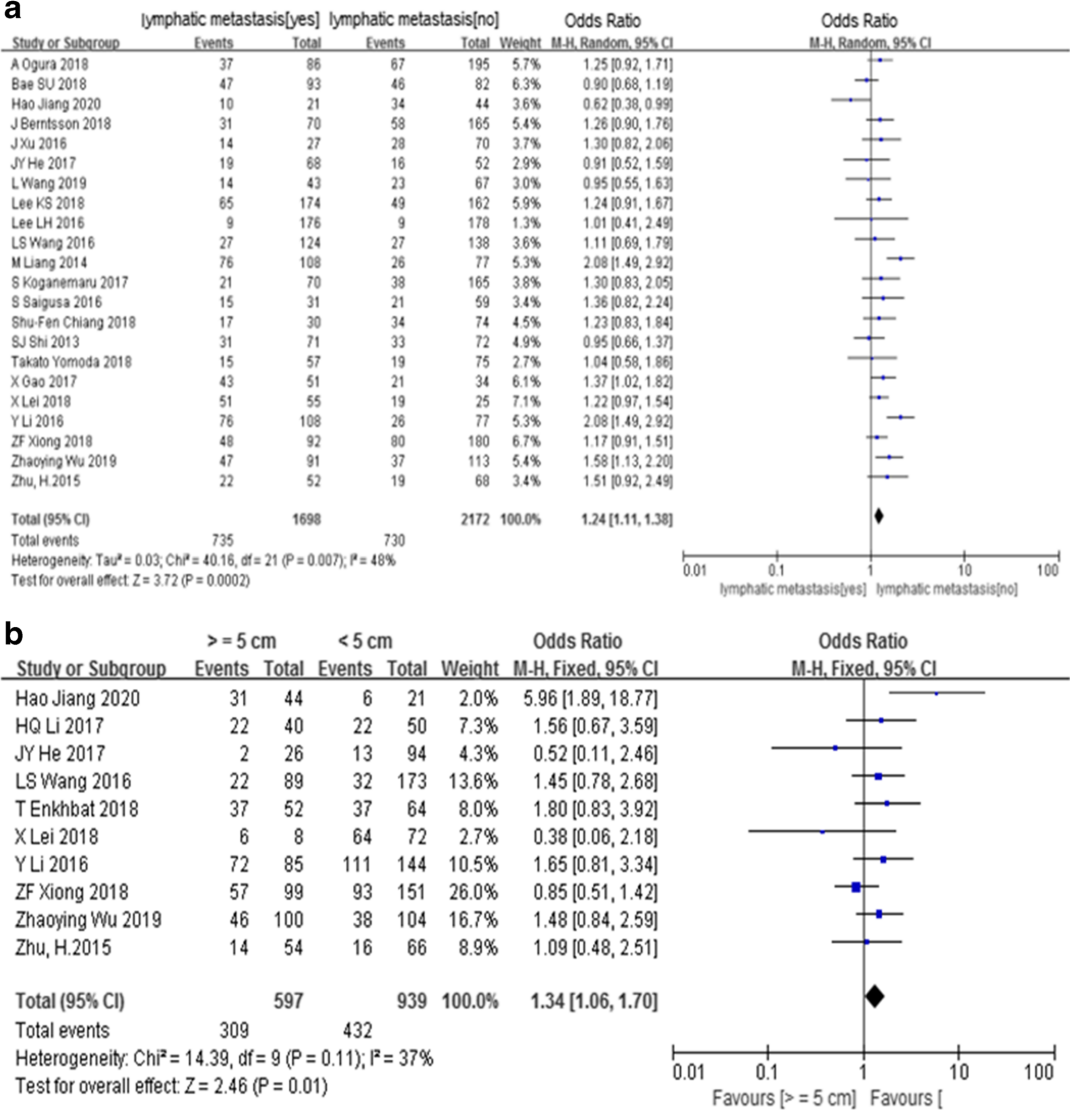
Meta-analysis between PD-L1 expression and lymphatic metastasis (**a**) and tumor diameter (**b**)

**Fig. 3:**
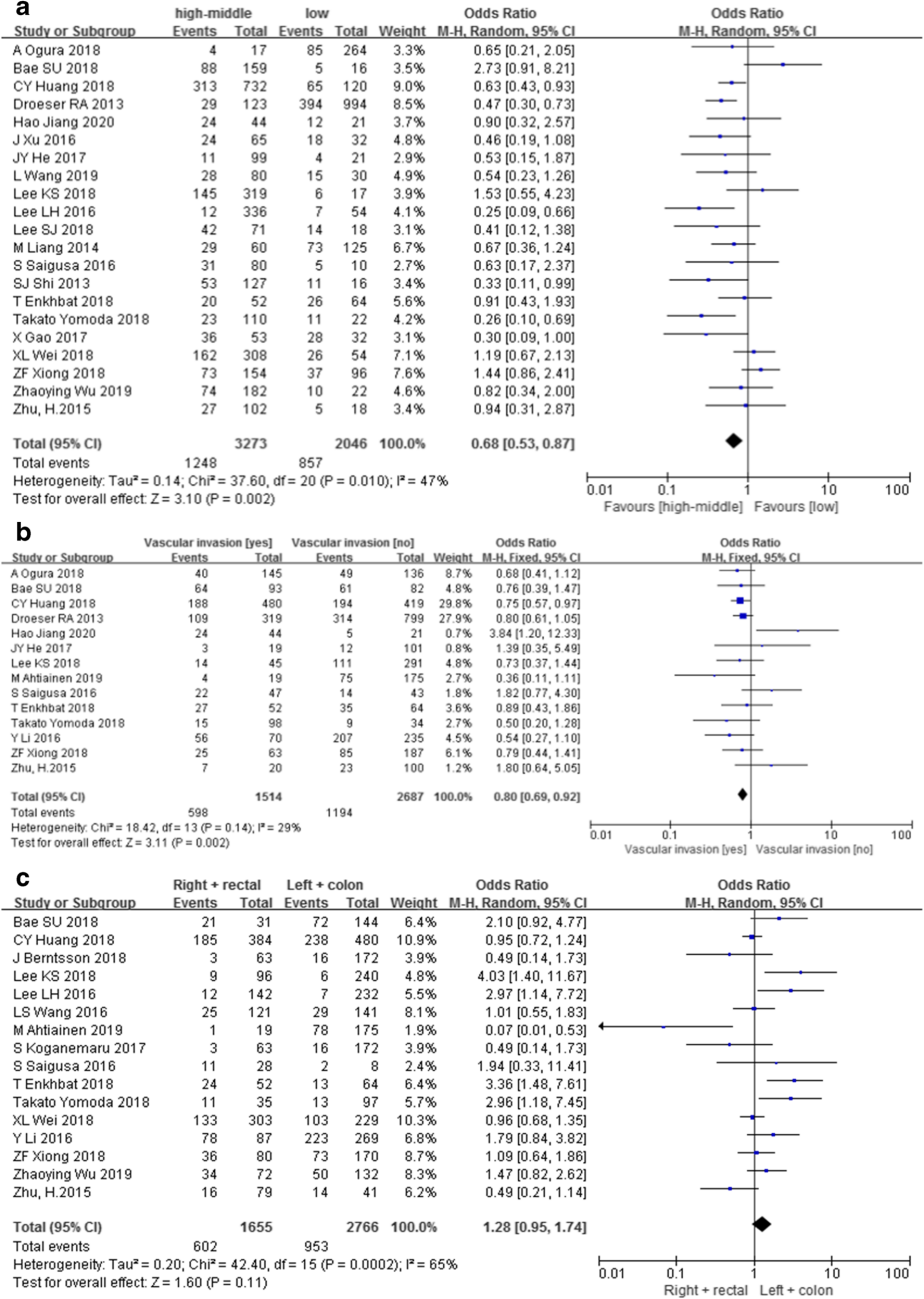
Meta-analysis between PD-L1 expression and differentiation (**a**) and vascular invasion (**b**) and tumor location (**c**)

The pooled OR indicated that there were significant positive correlations between PD-L1 expression and lymphatic metastasis (yes/no; *n* = 22; 3870 patients; OR = 1.24, 95% CI (1.11, 1.38), *Z* = 3.72, *P* < 0.05; *I*^2^ = 48%, *P* < 0.1) (Fig. 2a) and tumor diameter (≥ 5 cm/< 5 cm; *n* = 10; 1536 patients; OR = 1.34, 95% CI (1.06, 1.70), *Z* = 2.46, *P* < 0.05; *I*^2^ = 37%, *P* = 0.11) (Fig. 2b), but negative correlation with differentiation (high–middle/low; *n* = 21; 5319 patients; OR = 0.68, 95% CI (0.53, 0.87), *Z* = 3.10, *P* < 0.05; *I*^2^ = 47%, *P* < 0.1) (Fig. 3a) and vascular invasion (yes/no; *n* = 14; 4201 patients; OR = 0.80, 95% CI (0.69, 0.92), *Z* = 3.11, *P* < 0.05; *I*^2^ = 29%, *P* = 0.14) (Fig. 3b). However, there were no significant correlations found between PD-L1 expression and sex (male/female; *n* = 29; 8043 patients; OR = 0.94, 95% CI (0.85, 1.04), *Z* = 1.16, *P* > 0.05; *I*^2^ = 11%, *P* = 0.29) (Fig. S1A), age (≥ 60/<60 years old; *n* = 21; 4095 patients; OR = 0.96, 95% CI (0.84, 1.10), *Z* = 0.54, *P* > 0.05; *I*^2^ = 24%, *P* = 0.15) (Fig. S1B), TNM stage (III–IV/I–II; *n* = 23; 5108 patients; OR = 1.11, 95% CI (0.86, 1.43), *Z* = 0.81, *P* > 0.05; *I*^2^ = 57%, *P* < 0.1) (Fig. S2A), tumor location (right + rectal/left + colon; *n* = 16; 4421 patients; OR = 1.28, 95% CI (0.95, 1.74), *Z* = 1.60, *P* > 0.05; *I*^2^ = 65%, *P* < 0.1) (Fig. 3c), infiltration degree (3–4/1–2; *n* = 10; 1837 patients; OR = 0.82, 95% CI (0.64, 1.06), *Z* = 1.52, *P* > 0.05; *I*^2^ = 19%, *P* = 0.27) (Fig. S2B), and distant metastasis (yes/no; *n* = 10; 2486 patients; OR = 1.13, 95% CI (0.87, 1.47), *Z* = 0.91, *P* > 0.05; *I*^2^ = 30%, *P* = 0.18) (Fig. S2C).

### Correlation between PD-L1 expression and the prognostic parameters (OS, DFS, and PFS)

**Fig. 4:**
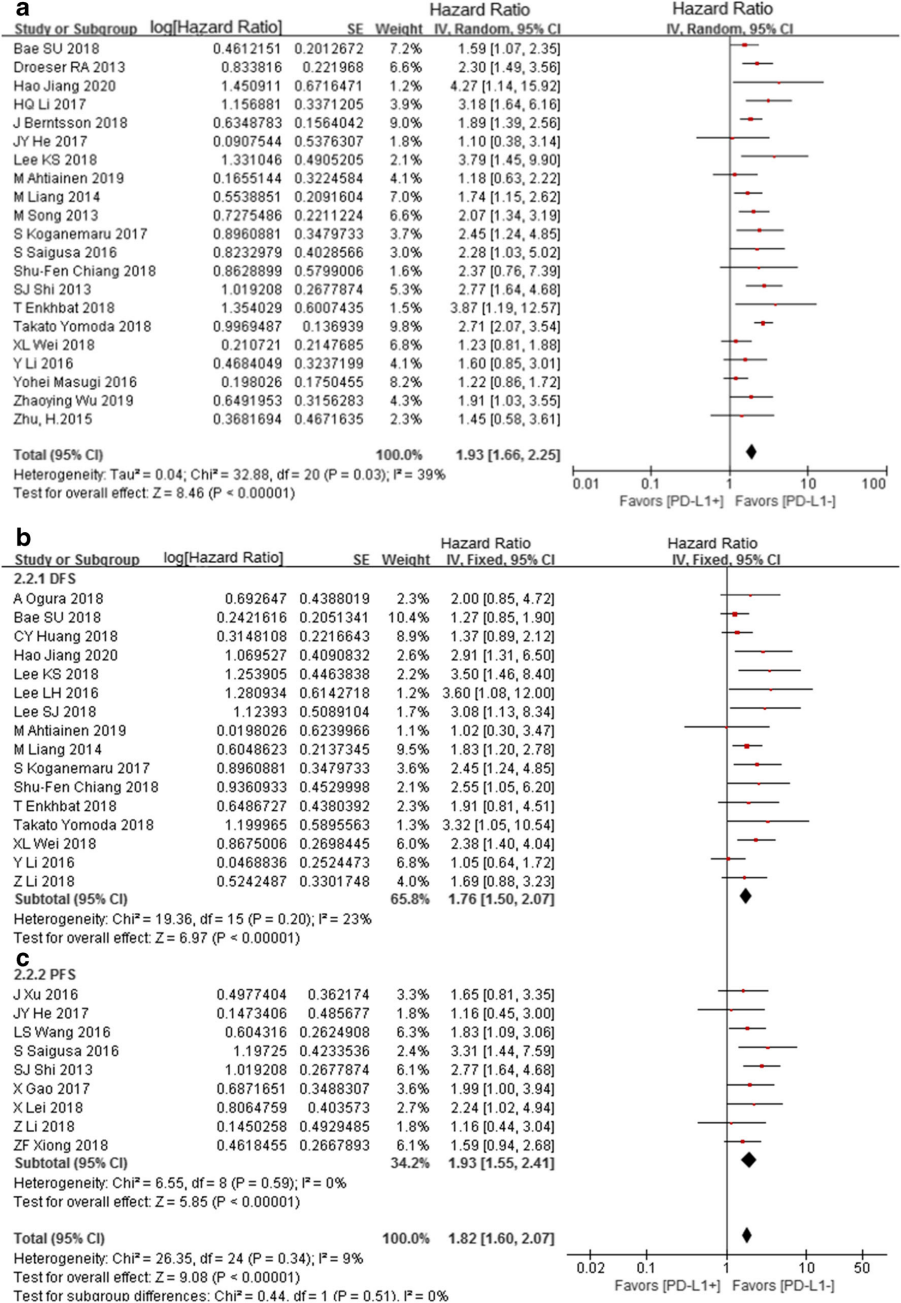
Meta-analysis of PD-L1 expression on OS (**a**), DFS (**b**), and PFS (**c**)

Twenty studies provided the OS parameters. As weak heterogeneity existed (*I*^2^ = 39%, *P* = 0.03), the random effects model was used. Meta-analysis showed that OS was significantly associated with PD-L1 expression in CRC patients (*n* = 21; HR = 1.93, 95% CI (1.66, 2.25), *Z* = 8.46, *P* < 0.05) (Fig. 4a).

Sixteen studies provided the DFS parameters. Results showed that DFS was significantly associated with PD-L1 expression in CRC patients (*n* = 16; HR = 1.76, 95% CI (1.50, 2.07), *Z* = 6.97, *P* < 0.05; *I*^2^ = 23%, *P* = 0.20) (Fig. 4b).

Nine studies provided the PFS parameters. Results showed that PFS was significantly associated with PD-L1 expression in CRC patients (*n* = 9; HR = 1.82, 95% CI (1.60, 2.07), *Z* = 5.85, *P* < 0.05; *I*^2^ = 0%, *P* = 0.59) (Fig. 4c).

### Subgroup analysis on OS under different treatment methods

**Fig. 5:**
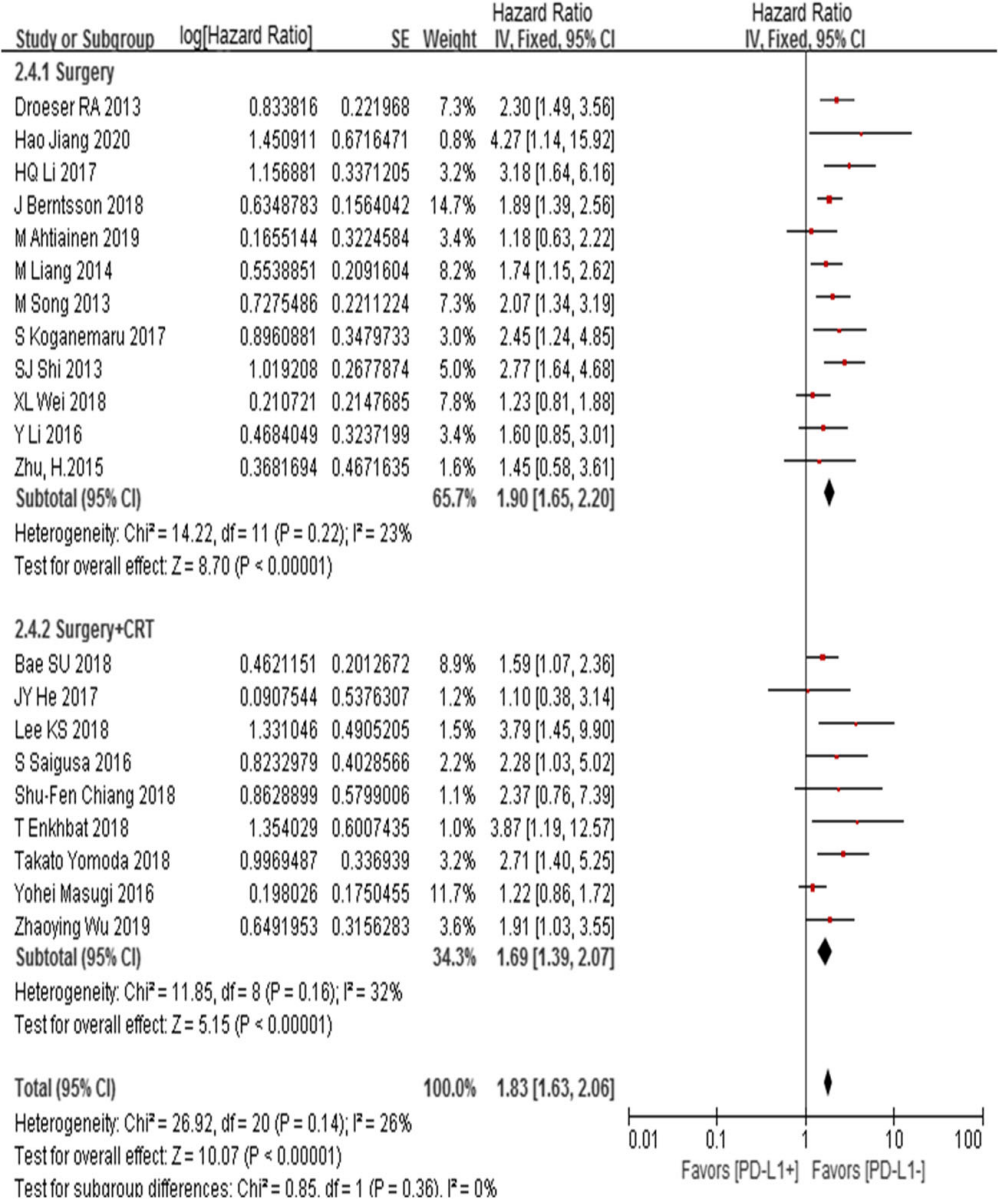
Subgroup analysis of PD-L1 expression on OS by using different treatment methods

Results were as follows: (1) surgery: PD-L1 expression was significantly associated with OS (*n* = 12; HR = 1.90, 95% CI (1.65, 2.20), *Z* = 8.70, *P* < 0.05; *I*^2^ = 23%, *P* = 0.22); (2) surgery + CRT: PD-L1 expression was significantly associated with OS (*n* = 9; HR = 1.69, 95% CI (1.39, 2.07), *Z* = 5.15, *P* < 0.05; *I*^2^ = 32%, *P* = 0.16) (Fig. 5).

### Meta-regression analysis

Meta-regression analysis confirmed that PD-L1 expression was to be correlated with OS (HR = 1.95, 95% CI (1.92, 3.98)) and DFS (HR = 2.14, 95% CI (0.73, 4.52)). And the prognosis of patients with surgery treatment alone was worse than that of surgery combined with CRT. Patients with distant metastasis had a poor prognosis (Table 3).

**Table 3:** HR and 95% CI in meta-regression analysis for CRC prognosis

Prognosis	Variables	HR	Standard error	*Z*	*P*	95% CI
OS	Distant metastasis (no/yes)	3.22	1.11	2.91	< 0.05	[1.05, 5.39]
	Treatment methods (surgery/surgery + CRT)	1.05	0.03	41.57	< 0.05	[1.00, 1.10]
	PD-L1 (negative/positive)	1.95	0.01	3.98	< 0.05	[1.92, 3.98]
DFS	Treatment methods (surgery/surgery + CRT)	0.84	0.30	2.75	< 0.05	[0.24, 1.43]
	PD-L1 (negative/positive)	2.14	0.21	3.77	< 0.05	[0.73, 4.52]

Sex, age, differentiation, lymphatic metastasis, infiltration degree, distant metastasis, tumor diameter, vascular invasion, TNM stage, tumor type, tumor location, PD-L1 expression, and treatment methods were used as adjustment factors in meta-regression analysis

### Sensitivity analysis

Sensitivity analysis on OS, DFS, and PFS indicated that after excluding any single study individually, there was no separate study that significantly affected HR and 95% CI, suggesting that the results of this meta-analysis were stable (Fig. S3).

### Publication bias

**Fig. 6:**
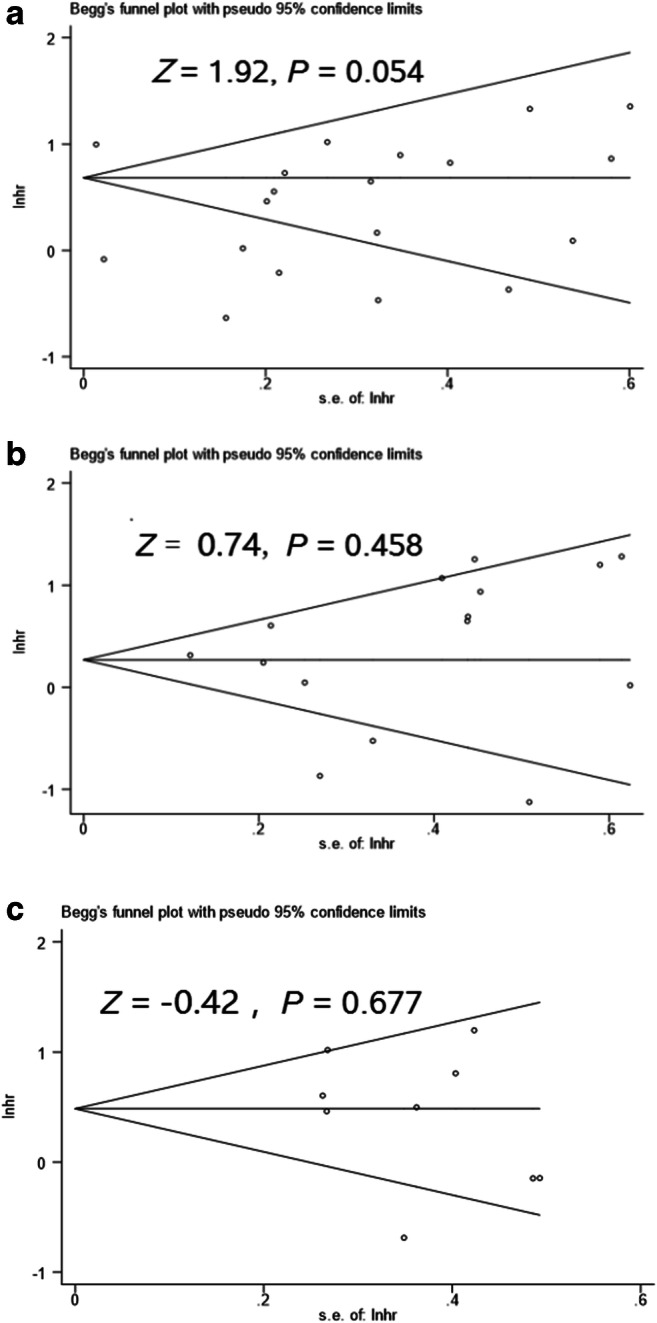
Begg’s funnel plot for OS (**a**), DFS (**b**), and PFS (**c**) publication bias in the included studies

Results of Begg’s test suggested that there may be no publication bias among studies for OS, DFS, and PFS (all *P* > 0.05) (Fig. 6).

## Discussion

Studies reported that the PD-1/PD-L1 pathway has become a promising therapeutic target for various human malignancies [17, 18, 57–60]. Nonetheless, the correlation between PD-L1 expression and clinicopathological features [26, 30] and the prognosis of CRC patients are still controversial [36, 51]. Therefore, this study comprehensively searched the literature to solve the above-existing controversies in order to draw more reliable conclusions.

Data of our meta-analysis from 32 studies (8823 CRC patients), the largest to date, indicated that PD-L1 expression was significantly positively correlated with lymphatic metastasis and tumor diameter, but negatively correlated with differentiation and vascular invasion. However previous meta-analysis found that PD-L1 expression was correlated with tumor stage [21] and gender [22] and tumor location [23], which results were unreliable due to high heterogeneity (all *I*^2^ > 70%) [21–23] and the incorrect analytical model (all selected the fixed effects model that is available for *I*^2^ < 50%) [21–23]. In this study, the random effects model was selected for TNM stage and tumor location because of mild heterogeneity (*I*^2^ = 57% for TNM stage and *I*^2^ = 65% for tumor location).

In univariate analysis, PD-L1 was correlated with poor prognosis of CRC in this study, which was similar to the results of previous meta-analysis [20–23]. However, high heterogeneity existed in our study and those meta-analyses [20–23]. Furthermore, in subgroup analysis based on treatment, we found that the degree of statistical heterogeneity reduced both in subgroup for OS (Fig. 5). It meant that the treatment method was the source of heterogeneity for OS. In order to control other confounders, meta-analysis should be necessary to analyze the independent role of PD-L1 on CRC prognosis. We found that PD-L1 expression independently predicted a poor prognostic outcome with meta-regression analysis. Previous meta-analysis made a contradictory conclusion by univariate analysis [20–23]. Meta-regression analysis can get a more reliable and accurate outcome after adjusting confounders including clinicopathological features and treatment methods that influence the CRC prognosis.

In our sensitivity analysis, none of the inclusions and exclusions of specific studies one by one materially changed the results of the primary meta-analysis; it suggested that the results of this meta-analysis were stable.

From the perspective of publication bias, Begg’s test on OS, DFS, and PFS found that there was no significant publication bias that existed among included studies, and the results of this study were relatively reliable.

Despite some positive findings from this meta-analysis, two limitations still existed to our study. Firstly, although Chinese and English studies were included in this meta-analysis, language bias still existed. Secondly, although the literature screening was carried out with a strict search strategy, a small number of literatures including gray literature and conference literature may still be missing.

## Conclusions

In summary, PD-L1 expression was significant correlated with lymphatic metastasis, tumor diameter, differentiation, and vascular invasion, and could act as an independently poor prognostic factor for CRC.

## Electronic supplementary material

ESM 1 (PDF 491 kb)

## Data Availability

The data supporting this meta-analysis are from previously published studies, which have been cited. The processed data are available from the first author (wangshuxialucky@163.com) upon request.
