# Supplementary material for: Clinicopathological and prognostic significance of PD-L1 expression in colorectal cancer: a meta-analysis
Source: Int J Colorectal Dis. 2020 Sep 10;36(1):117–30. doi: 10.1007/s00384-020-03734-4 (PMC7782388; doi:10.1007/s00384-020-03734-4)
Supplement: Supplementary file 1 — (PDF 491 kb) [file 384_2020_3734_MOESM1_ESM.pdf]

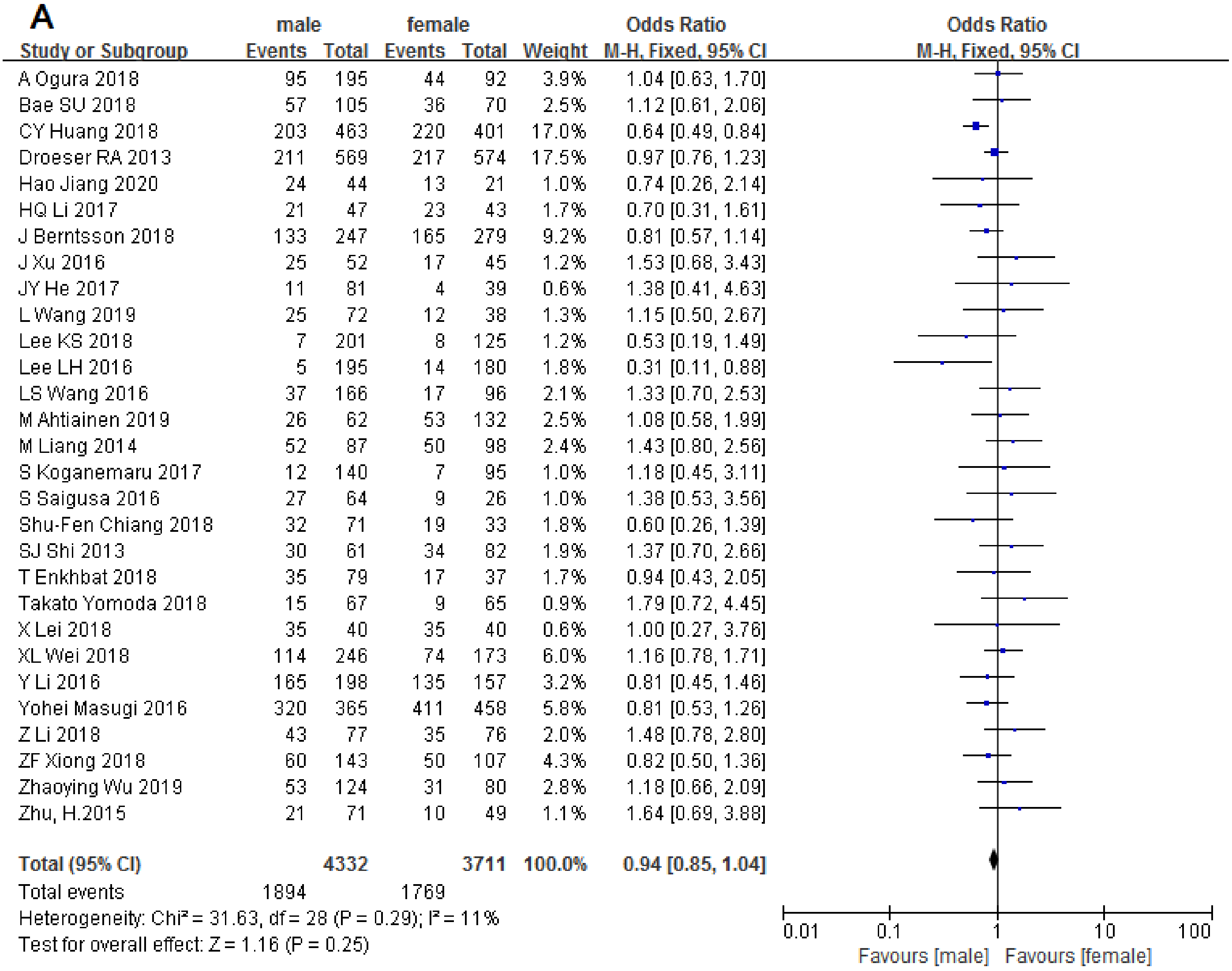

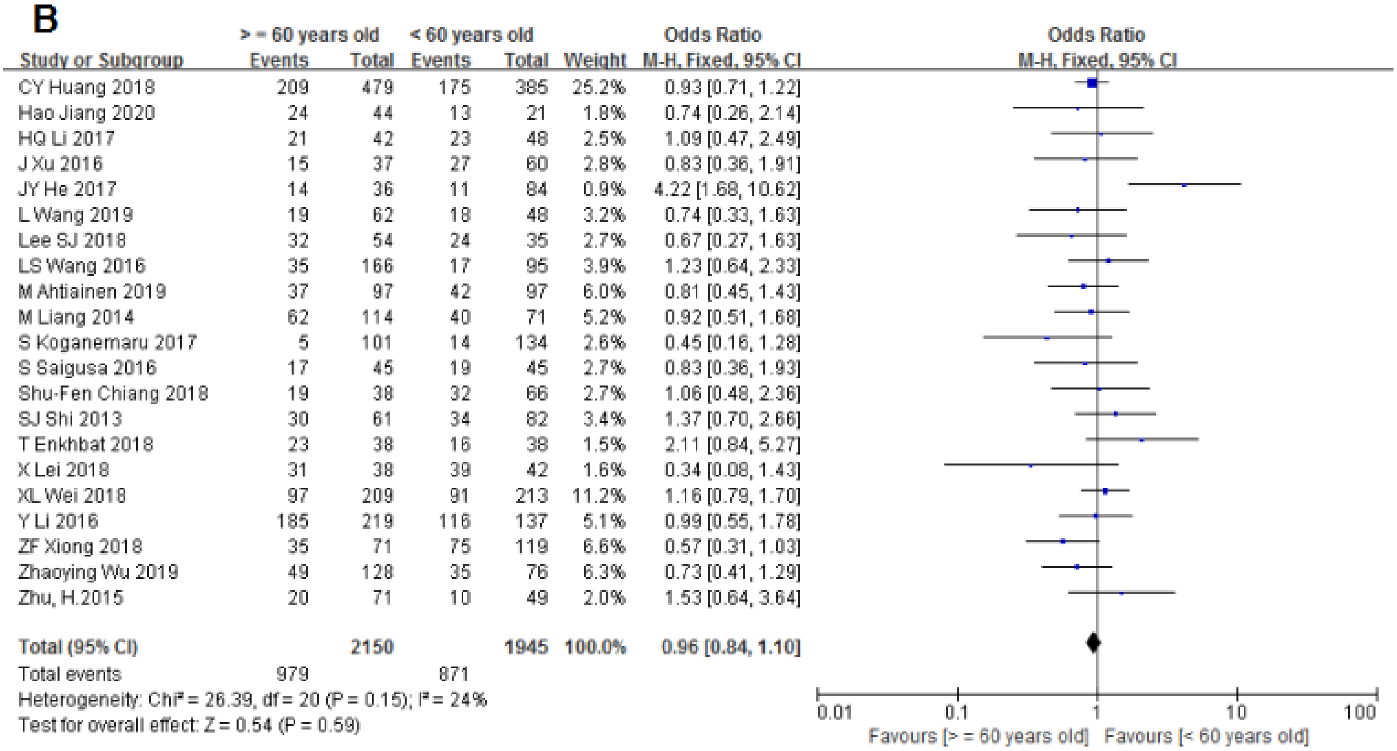

Figure.S1 Meta-analysis between PD-L1 expression and sex (A) and age (B)

A

Study or Subgroup	III-IV Events	III-IV Total	I-II Events	I-II Total	Weight	Odds Ratio M-H, Random, 95% CI
A Ogura 2018	115	228	28	60	5.4%	1.16 [0.66, 2.06]
Bae SU 2018	48	94	45	81	5.3%	0.83 [0.46, 1.52]
CY Huang 2018	180	408	252	456	6.9%	0.64 [0.49, 0.84]
HQ Li 2017	22	34	32	46	3.7%	0.80 [0.31, 2.06]
J Xu 2016	17	27	35	60	3.7%	1.21 [0.48, 3.09]
JY He 2017	81	91	15	29	3.5%	7.56 [2.83, 20.16]
L Wang 2019	15	47	22	63	4.3%	0.87 [0.39, 1.95]
Lee KS 2018	91	174	83	159	6.1%	1.00 [0.65, 1.54]
Lee SJ 2018	19	32	37	57	3.9%	0.79 [0.32, 1.93]
LS Wang 2016	26	124	28	138	5.3%	1.04 [0.57, 1.90]
M Ahtiainen 2019	6	70	16	124	3.5%	0.63 [0.24, 1.70]
M Liang 2014	75	123	27	72	5.3%	2.60 [1.43, 4.74]
S Koganemaru 2017	69	182	29	53	5.2%	0.51 [0.27, 0.94]
S Saigusa 2016	18	35	28	55	4.1%	1.02 [0.44, 2.38]
Shu-Fen Chiang 2018	50	100	1	4	1.0%	3.00 [0.30, 29.83]
SJ Shi 2013	27	78	37	65	4.9%	0.40 [0.20, 0.79]
T Enkhbat 2018	27	57	25	59	4.6%	1.22 [0.59, 2.55]
X Gao 2017	46	52	26	33	2.8%	2.06 [0.63, 6.80]
X Lei 2018	47	54	19	26	2.9%	2.47 [0.76, 8.01]
Y Li 2016	242	288	78	87	4.5%	0.61 [0.28, 1.30]
Yohei Masugi 2016	309	339	370	420	5.9%	1.39 [0.86, 2.24]
Zhaoying Wu 2019	49	98	35	106	5.4%	2.03 [1.15, 3.57]
Zhu, H.2015	28	103	2	17	2.0%	2.80 [0.60, 13.03]
**Total (95% CI)**		**2838**		**2270**	**100.0%**	**1.11 [0.86, 1.43]**
Total events	1607		1270			

Heterogeneity: Tau² = 0.23; Chi² = 66.73, df = 22 (P < 0.00001); I² = 57%

Test for overall effect: Z = 0.81 (P = 0.42)

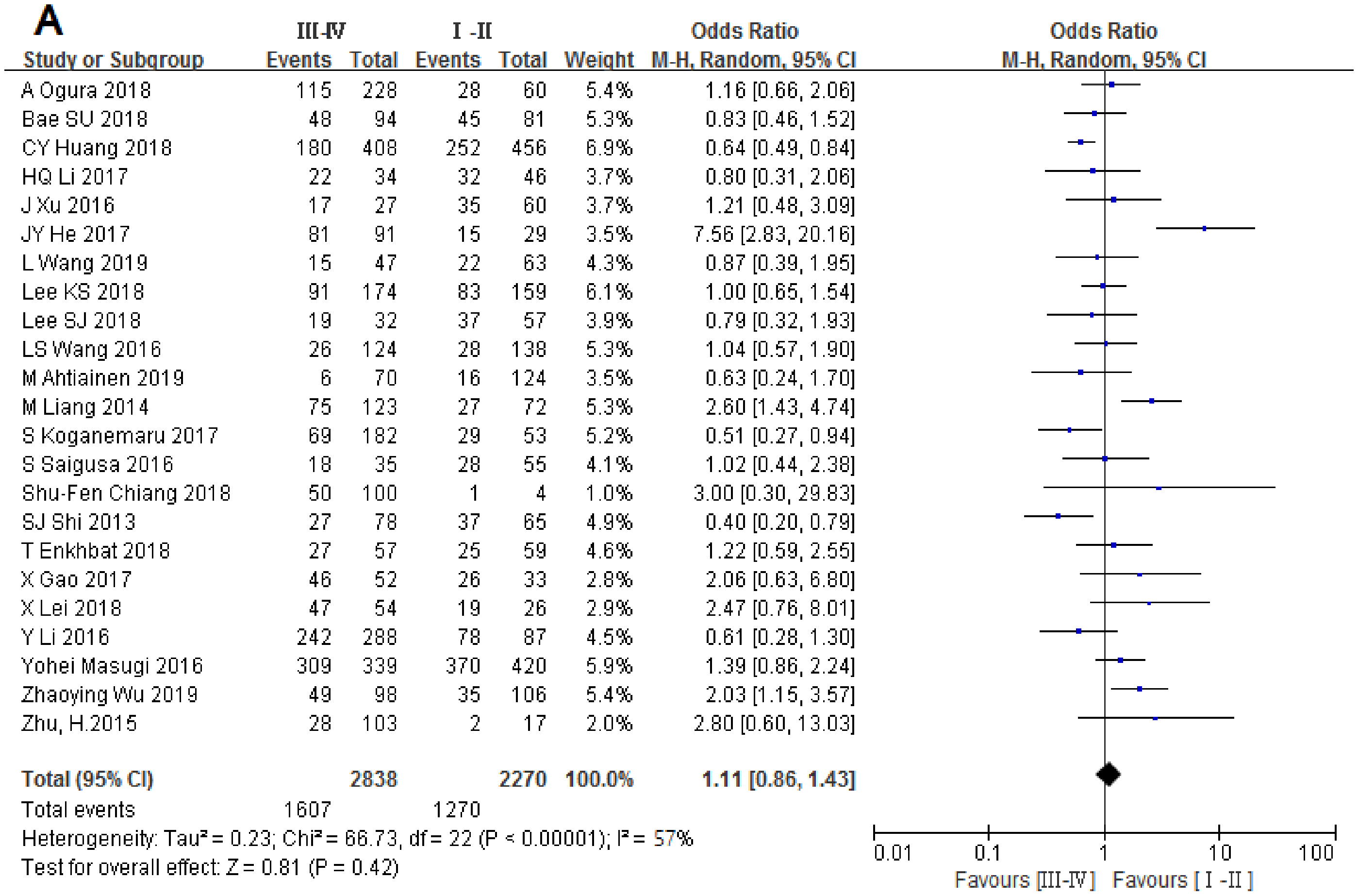

B

Study or Subgroup	infiltration degree (3-4) Events	infiltration degree (3-4) Total	infiltration degree (1-2) Events	infiltration degree (1-2) Total	Weight	Odds Ratio M-H, Fixed, 95% CI
A Ogura 2018	39	133	34	104	19.8%	0.85 [0.49, 1.49]
Bae SU 2018	73	144	20	31	11.9%	0.57 [0.25, 1.26]
J Xu 2016	20	51	22	46	10.3%	0.70 [0.31, 1.58]
L Wang 2019	33	85	14	25	9.7%	0.50 [0.20, 1.23]
LS Wang 2016	51	249	5	15	5.5%	0.52 [0.17, 1.57]
S Saigusa 2016	23	50	13	40	5.7%	1.77 [0.75, 4.20]
T Enkhbat 2018	48	52	60	64	3.0%	0.80 [0.19, 3.37]
Takato Yomoda 2018	21	83	13	39	9.7%	0.68 [0.30, 1.55]
XL Wei 2018	166	382	22	40	16.5%	0.63 [0.33, 1.21]
Zhaoying Wu 2019	72	164	12	40	7.9%	1.83 [0.87, 3.84]
**Total (95% CI)**		**1393**		**444**	**100.0%**	**0.82 [0.64, 1.06]**
Total events	546		215			

Heterogeneity: Chi² = 11.15, df = 9 (P = 0.27); I² = 19%

Test for overall effect: Z = 1.52 (P = 0.13)

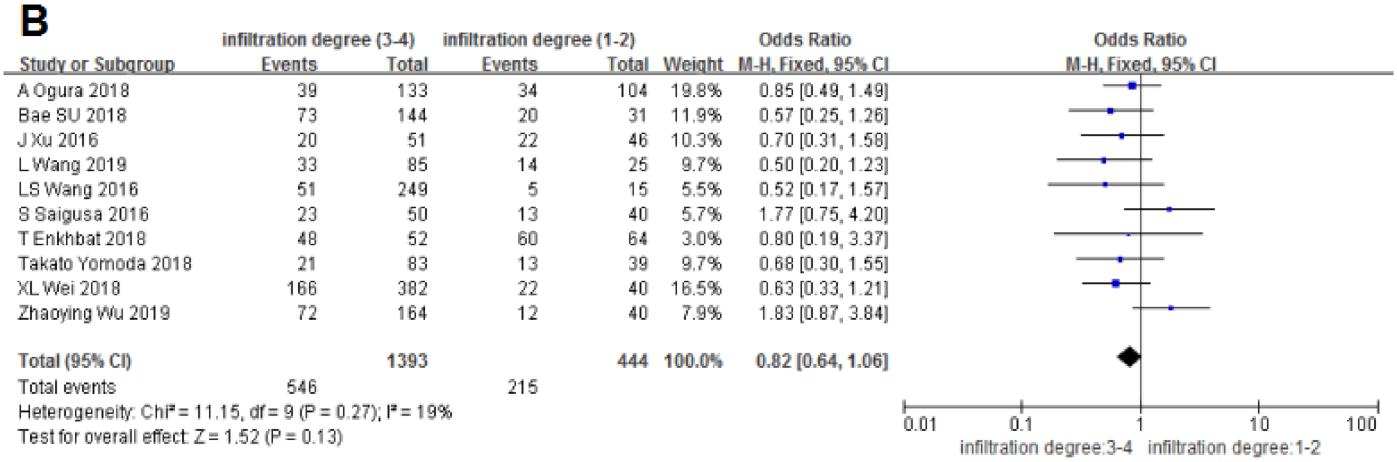

C

Study or Subgroup	Distant metastasis [yes] Events	Distant metastasis [yes] Total	Distant metastasis [no] Events	Distant metastasis [no] Total	Weight	Odds Ratio M-H, Fixed, 95% CI
J Berntsson 2018	35	87	259	432		Not estimable
L Wang 2019	5	12	32	98	3.9%	1.47 [0.43, 5.00]
Lee KS 2018	17	32	145	299	12.6%	1.20 [0.58, 2.50]
Shu-Fen Chiang 2018	14	20	37	84	4.1%	2.96 [1.04, 8.46]
X Lei 2018	9	10	60	70	1.4%	1.50 [0.17, 13.16]
XL Wei 2018	26	68	162	354	30.8%	0.73 [0.43, 1.25]
Y Li 2016	90	111	211	245	23.8%	0.69 [0.38, 1.25]
Yohei Masugi 2016	111	118	568	641	10.0%	2.04 [0.91, 4.54]
Zhaoying Wu 2019	10	19	74	185	6.2%	1.67 [0.65, 4.30]
Zhu, H.2015	9	19	45	101	7.2%	1.12 [0.42, 2.99]
**Total (95% CI)**		**409**		**2077**	**100.0%**	**1.13 [0.87, 1.47]**
Total events	291		1334			

Heterogeneity: Chi² = 11.39, df = 8 (P = 0.18); I² = 30%

Test for overall effect: Z = 0.91 (P = 0.36)

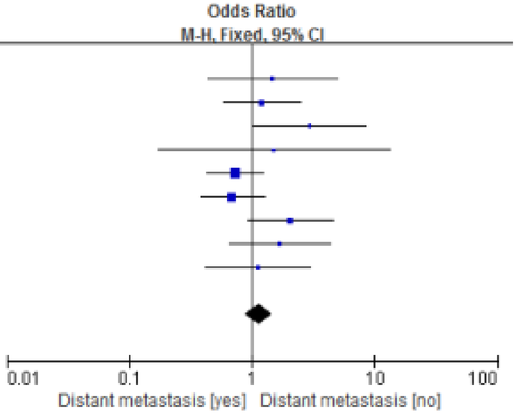

Figure.S2 Meta-analysis between PD-L1 expression and TNM stage (A) and infiltration degree (B) and distant metastasis (C)

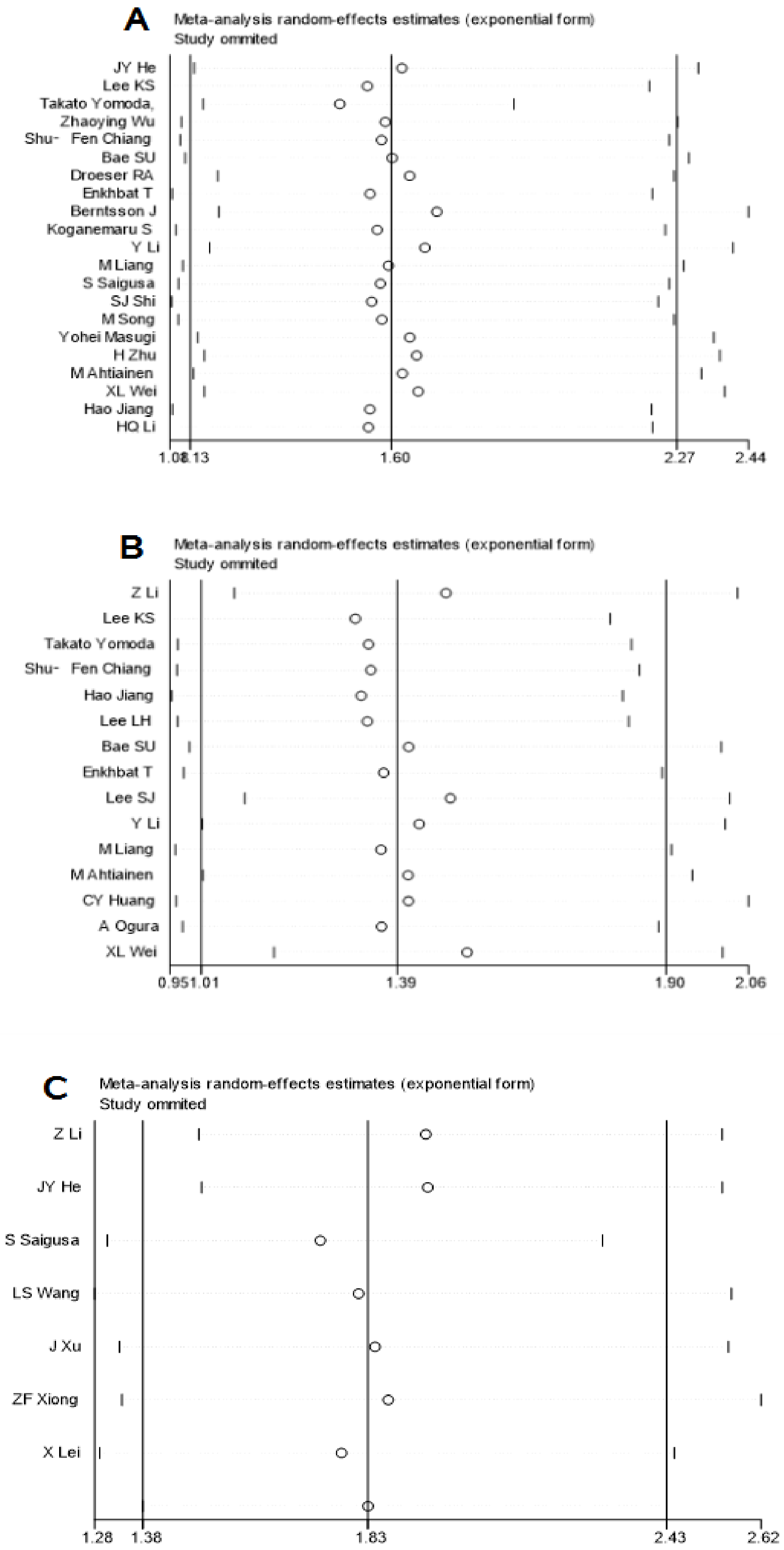

Figure.S3 Sensitivity analysis on OS (**A**), DFS (**B**) and PFS (**C**)
